# Transcriptomic Analysis Reveals Functional Interaction of mRNA-lncRNA-miRNA in *Trachinotus ovatus* Infected by *Cryptocaryon irritans*

**DOI:** 10.3390/ijms242115886

**Published:** 2023-11-01

**Authors:** Baosuo Liu, Lize San, Huayang Guo, Kecheng Zhu, Nan Zhang, Jingwen Yang, Bo Liu, Jilun Hou, Dianchang Zhang

**Affiliations:** 1 Key Laboratory of South China Sea Fishery Resources Exploitation and Utilization, Ministry of Agriculture and Rural Affairs, South China Sea Fisheries Research Institute, Chinese Academy of Fishery Sciences, Guangzhou 510300, China; liubaosuo343@163.com (B.L.); erkelb@126.com (B.L.); 2 Guangdong Provincial Engineer Technology Research Center of Marine Biological Seed Industry, Guangzhou 510300, China; 3 Hebei Key Laboratory of the Bohai Sea Fish Germplasm Resources Conservation and Utilization, Beidaihe Central Experiment Station, Chinese Academy of Fishery Sciences, Qinhuangdao 066100, China; 4 Sanya Tropical Fisheries Research Institute, Sanya 572000, China

**Keywords:** ceRNA network, non-coding RNA, *Trachinotus ovatus*, *Cryptocaryon irritans*, immune response

## Abstract

The skin of *Trachinotus ovatus* is a crucial component of the mucosal immune system and serves as the primary site of infection by *Cryptocaryon irritans*. In order to investigate the significant role of skin in *C. irritans* infection, a comprehensive transcriptome analysis was conducted on skin tissues from the infection group, infection-adjacent group, and infection group compared with the infection-adjacent group (ATT_vs_PER, ADJ_vs_PER, ATT_vs_ADJ). This study identified differentially expressed long non-coding RNAs (DE lncRNAs), microRNAs (DE miRNAs), and differentially expressed genes (DEGs). The prediction of lncRNA target genes was accomplished by utilizing positional relationship (co-location) and expression correlation (co-expression) with protein-coding genes. Subsequently, functional enrichment analysis was conducted on the target genes of differentially expressed lncRNAs, revealing their involvement in signaling pathways such as tight junction, MAPK, and cell adhesion molecules. This study describes the regulatory network of lncRNA-miRNA-mRNA in *T. ovatus* skin tissue infected with *C. irritans*. Functional prediction analysis showed that differentially expressed lncRNA and miRNA may regulate the expression of immune genes such as interleukin-8 (*il8*) to resist the infection of *C. irritans*. Conducting additional research on these non-coding RNAs will facilitate a deeper understanding of their immune regulatory function in *T. ovatus* during *C. irritans* infection. The study of non-coding RNA in this study laid a foundation for revealing the molecular mechanism of the immune system of *T. ovatus* to respond to the infection of *C. irritans*. It provided a choice for the molecular breeding of *Trachinotus ovatus* against *C. irritans*.

## 1. Introduction

*Cryptocaryon irritans* disease is a lethal affliction that arises from the parasitization of marine teleost fish by *C. irritans*. Typically, this parasite infests the gills, skin, and fins of fish. The life cycle of *C. irritans* includes four stages: trophont, protomont, tomont, and theront. Upon the development of theronts into trophonts on the body surface, small white spots manifest on the body surface, commonly referred to as “white spot disease” [1]. *C. irritans* can induce organ damage and host dysfunction, including dyspnea, aberrant behavior, epithelial hyperplasia, and mechanical harm to fish, and may readily lead to secondary bacterial infections, which can lead to host death when the *C. irritans* causes infection in large numbers [2,3]. *C. irritans* exhibits a high degree of infectivity across a broad spectrum of marine fish species, with no discernible preference for individual fish size. Notably, this pathogen is capable of infecting numerous marine teleosts, including but not limited to *Larimichthys crocea*, *Lateolabrax japonicus*, *Nibea albiflora*, and *Rachycentron canadus*. *C. irritans* has a broad thermal tolerance range, lack of intermediate hosts, and a brief life cycle [4,5,6,7]. *C. irritans* can reproduce rapidly after infection, and there is no effective treatment for *C. irritans* infection, eventually leading to many fish deaths, resulting in huge economic losses to the aquaculture industry.

Several techniques, such as RT-PCR and RNA sequencing, have been employed to elucidate the molecular mechanisms underlying the response of *T. ovatus* to *C. irritans*. The entire transcriptome encompasses all transcripts that are expressed by a particular tissue or cell at a specific time and location, comprising both coding RNA (mRNA) and non-coding RNA, the latter of which predominantly comprises micro-RNA (miRNA) and long non-coding RNA (lncRNA). In contrast to conventional transcriptome sequencing, whole transcriptome sequencing surpasses the limitations of analyzing a single RNA and enables the investigation of the regulation and interaction between RNAs. LncRNA exerts its regulatory influence on target genes through cis-regulation and trans-regulation, primarily at the transcription, post-transcription, translation, and epigenetic levels [8]. Conversely, miRNA operates at the post-transcriptional level by binding to the 3′UTR or 5′UTR of target mRNA and is a small non-coding RNA that plays a crucial role in regulating various biological processes in fish [9,10]. Micro RNAs regulate gene expression at the post-transcriptional level by binding to the non-translation region of the 3′ end of mRNA, while lncRNAs act as the endogenous competing RNAs of miRNAs, inhibiting the binding of miRNAs and mRNA and playing a role in immune regulation. With the development of sequencing technology, detecting miRNAs and lncRNAs expression patterns in tissues has become an important technology for studying fish immune gene expression regulation by non-coding RNA.

The regulation of immune genes by miRNA has been extensively investigated in fish, with previous studies demonstrating the differential expression of miRNAs in fish infected with viruses [11]. In a study by Zhang et al. [12], miRNA sequencing analysis of spleens from Japanese flounder (*Paralichthys olivaceus*) that were infected with megalocytivirus at various time points revealed 121 miRNAs that were differentially expressed. This finding suggests that *megalocytivirus* infection may activate the miRNA regulation of genes related to immune response, signal transduction, and apoptosis. Viral hemorrhagic septicemia virus (VHSV) is another virus that causes massive mortality in captive fish. Najib et al. [13] found in a study of VHSV infection in olive flounder (*P. olivaceus*) that miRNAs affect the expression of immune-related genes such as complement and interleukins. In addition, studies have confirmed that there are differentially expressed miRNAs in the tissues of zebrafish (*Danio rerio*), common carp (*Cyprinus carpio*), Chinese tongue sole (*Cynoglossus semilaevis*), and Nile tilapia (*Oreochromis niloticus*) after bacterial infection [14,15,16,17]. LncRNAs are a group of RNA molecules that exceed 200 nucleotides in length and typically lack protein-coding capacity. They are known to modulate diverse cellular processes, including but not limited to disease, immunity, development, and cell proliferation. Notably, a study on three genetic strains of rainbow trout (*Oncorhynchus mykiss*) infected with *Flavobacterium psychrophilum* revealed significant positive and negative associations between the expression of certain lncRNAs and immune-related gene products. These findings suggest a potential role for lncRNAs in the regulation of immune responses to bacterial infections [18]. Boltana et al. [19] conducted a study on the expression profiles of liver, head, and kidney lncRNAs in Atlantic salmon (*Salmo salar*) during ISA virus (ISAV) infection. The study’s findings indicated the potential involvement of lncRNAs in regulating the host response during ISAV infection. Consequently, whole transcriptome analysis has emerged as a crucial approach to investigate fish immune regulation. Identifying differential transcripts and key gene regulatory networks through this method offers novel avenues for research and problem-solving.

The *T. ovatus*, commonly known as the golden pompano, is predominantly found in the coastal waters of Fujian, Guangdong, and Hainan in China and is a significant economic fish in the South China Sea. However, the intensification of cage culture and the consequent increase in breeding density have led to frequent outbreaks of diseases, particularly those caused by *C. irritans* infection. The severity of these diseases is such that they result in the massive mortality of *T. ovatus* and cause significant economic losses to the *T. ovatus* culture industry. The Mucosal immune system serves as the primary line of defense for fish, with scales providing an additional layer of protection. In the absence of scales, certain cells within the epidermis and dermis are responsible for maintaining the skin’s defensive capabilities. The skin plays a crucial role in immune protection by creating a barrier between the internal and external environments of the body and by secreting mucus and lysozyme. Nevertheless, the changes and regulatory mechanisms of immune gene expression patterns in the skin tissue of *T. ovatus* in response to *C. irritans* infection are still unclear. The immune system is being comprehended to a greater extent due to the swift progression of high-throughput technologies and bioinformatics analysis, leading to an increased significance of new ceRNA hypotheses. The significance of ncRNAs as finely tuned regulators of transcriptional responses is increasingly supported by mounting evidence. Most ncRNAs regulate the expression of numerous crucial mRNAs, and the intricate and interconnected ceRNA network between coding genes and ncRNA is complex. This study comprehensively analyzed the expression profiles of miRNAs, lncRNAs, and mRNAs in skin tissues. Following the identification of differentially expressed miRNAs, lncRNAs, and genes, functional enrichment analysis was conducted to predict key genes involved in immune-related pathways. Subsequently, a ceRNA network was established based on transcriptome analysis, which clarified the function of lncRNA-miRNA-mRNA interaction and analyzed the non-coding RNAs that regulate the expression of key genes, thereby providing novel insights into the immune response to *C. irritans* infection in *T. ovatus*. Screening key immune genes and regulating their non-coding RNAs can be applied to developing disease vaccines and breeding disease-resistant varieties.

## 2. Results

### 2.1. Reads Quality Control and Mapping to the Reference Genome

The RNA extracted from the skin tissue samples of both the infected and control groups was deemed suitable for sequencing. Subsequently, library construction and sequencing were performed, resulting in the generation of 80.79 million to 93.18 million raw reads. On average, 82.71 million effective reads were obtained per sample, with 86% of the reads successfully mapped to the reference genome of fish. Table 1 displays the quality control and mapping results of individual samples.

### 2.2. mRNA Differential Expression Analysis

**Figure 1:**
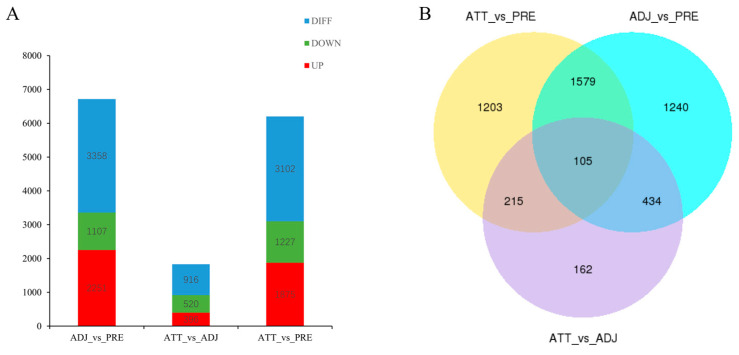
(**A**) Differentially expressed genes in the skin of infection group, infection-adjacent group, and infection group compared with infection-adjacent group (ATT_vs_PER, ADJ_vs_PER, ATT_vs_ADJ). (**B**) Venn diagram of the faltered differentially expressed genes of the ATT_vs_PER, ADJ_vs_PER, and ATT_vs_ADJ.

To identify the principal genes responsible for the resistance of fish skin tissue to *C. irritans* infection, the sequencing data underwent quality control and were subjected to differential gene screening and analysis using a *p* < 0.05 screening criterion. A total of 3102, 3358, and 916 differentially expressed genes were isolated from the skin tissue of the infected group (ATT_vs_PER), the adjacent group (ADJ_vs_PER), and the infected group compared with the adjacent group (ATT_vs_ADJ), respectively. Among these genes, 1875, 2251, and 396 were upregulated, while 1227, 1107, and 520 were downregulated. A Venn diagram presented a visual representation of the distinctively expressed genes among the three comparison groups. Specifically, the infection group and adjacent group exhibited 1579 differentially expressed genes, while the infected group and the infected group compared with the adjacent group displayed 215 differentially expressed genes (Figure 1A). Additionally, the adjacent group and the infected group compared with the adjacent group manifested 434 differentially expressed genes. Finally, the three comparison groups collectively exhibited 105 differentially expressed genes (Figure 1B).

### 2.3. Function Enrichment of Differentially Expressed mRNAs

In order to investigate the biological functions of DEG in greater detail, we utilized the KEGG database to map it and subsequently enriched it within significant pathways based on the entire transcriptome background. The DEGs were related to biological pathways including cellular processes, environmental information processing, genetic information processing, and metabolism. Through KEGG enrichment analysis, we discovered differential genes in the skin samples from the ATT_vs_PER, ADJ_vs_PER, and ATT_vs_ADJ groups. Notably, all differential genes in the ATT_vs_PER group were enriched in 150 signaling pathways. The observed phenomena include phagosome, purine metabolism, protein processing in the endoplasmic reticulum, cell cycle, pyrimidine metabolism, and endoplasmic reticulum. The cytokine-cytokine receptor interaction exhibits a greater diversity of gene enrichment. The differential genes in the ADJ_vs_PER group were enriched in 149 signaling pathways, including protein processing in the endoplasmic reticulum with ribosome, phagosome, lysosome, tight junction, and carbon metabolism, which were abundant in different genes. In the ATT_vs_ADJ group, a total of 118 signaling pathways were found to be enriched with differentially expressed genes. Notably, several pathways including ribosome, calcium signaling pathway, cardiac muscle contraction, focal adhesion, and regulation of actin cytoskeleton were enriched by distinct genes.

### 2.4. Target Gene Identification and Functional Analysis of DE lncRNAs

**Figure 2:**
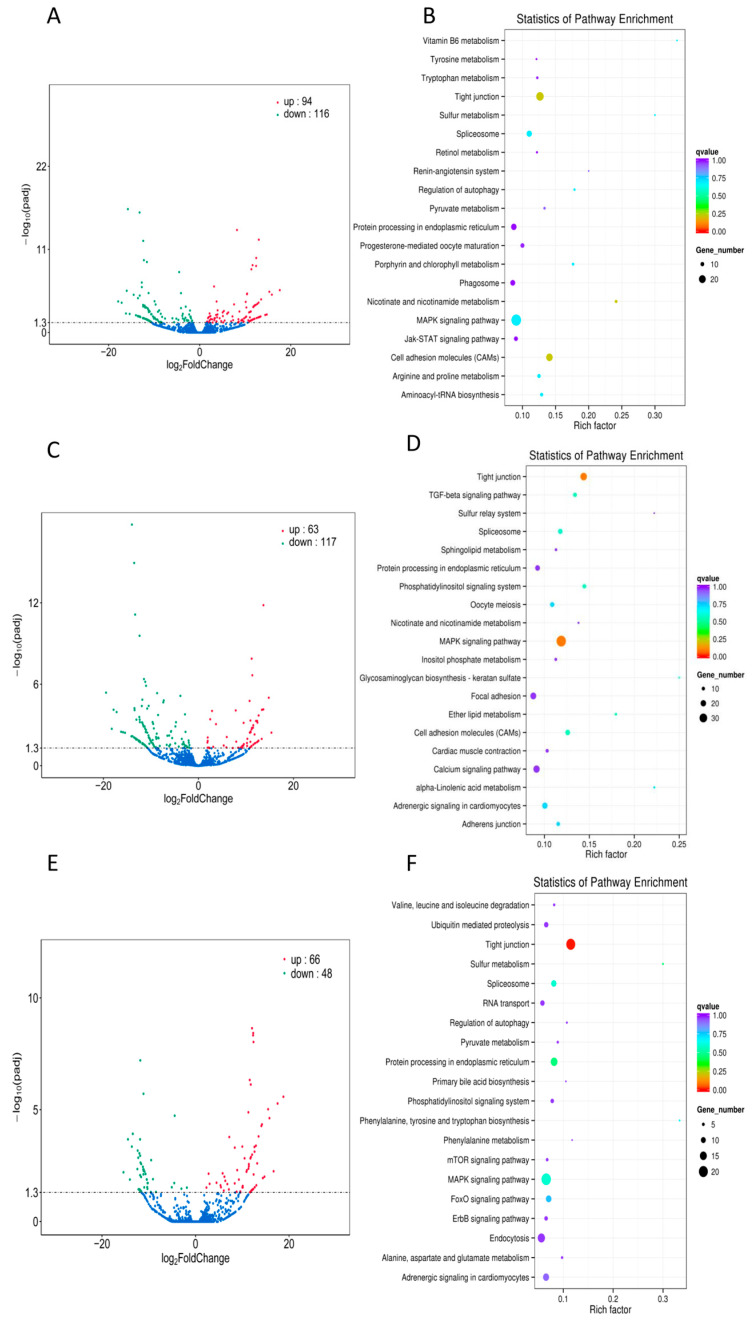
(**A**,**C**,**E**) Volcano plots from the differential expression lncRNAs of different groups (ATT_vs_PER, ADJ_vs_PER, ATT_vs_ADJ). The −log10 of the *p*adj value is used for the Y axis and the log2 of the fold change is used for the X axis. The red dots are upregulated lncRNAs and the green dots are downregulated lncRNAs. The blue dots were non-significant differentially expressed lncRNAs. (**B**,**D**,**F**) KEGG pathway analysis of the differentially expressed lncRNAs of different groups (ATT_vs_PER, ADJ_vs_PER, ATT_vs_ADJ). The vertical coordinates depict the various pathways, while the horizontal coordinates indicate the proportion of genes that exhibit significant differential expression within each respective pathway relative to the total number of genes within that pathway. The size of the circles corresponds to the number of genes enriched within each pathway, with larger circles denoting a more significant enrichment. The color of the circles signifies the level of significance of the enrichment, with shades closer to red indicating a higher degree of significance.

Before identifying lncRNA, transcriptional splicing was conducted, and known and novel mRNA with coding potential was filtered out. Subsequently, lncRNA was screened based on its unique structural characteristics. A total of 6571 lncRNAs were identified and categorized into three comparison groups: the infection group, the infection-adjacent group, and the infection group compared with the infection-adjacent group (ATT_vs_PER, ADJ_vs_PER, ATT_vs_ADJ). In skin tissues, 210, 180, and 114 differential lncRNAs were identified, with 94, 63, and 66 upregulated and 116, 117, and 48 downregulated, respectively (Figure 2A,C,E). Analysis of differentially expressed lncRNAs between groups revealed 72 differentially expressed lncRNAs in the ATT_vs_PER and ADJ_vs_PER comparison groups.

The prediction of the target genes for lncRNA involves co-location and co-expression. In order to perform a location-dependent target gene analysis, the position relationship between lncRNA and mRNA was considered, with a screening range of 100 Kb. Similarly, for the analysis of expression correlation target genes, the prediction was based on the expression correlation between lncRNA and mRNA, with a screening condition of an absolute correlation coefficient greater than 0.95. The co_location target gene prediction method identified 134 signaling pathways enriched with target genes predicted by all differentially expressed lncRNAs in the infection group (ATT_vs_PER). KEGG signal pathway enrichment analysis revealed that tight junction, MAPK, cell adhesion molecules, and other signal pathways exhibited more significant gene enrichment (Figure 2B).

Similarly, the infection-adjacent group (ADJ_vs_PER) demonstrated the enrichment of predicted target genes in 130 signaling pathways. KEGG signaling pathway enrichment analysis results indicate that several pathways, including tight junction, MAPK, focal adhesion, and calcium signaling pathways, were enriched with more genes. Figure 2D illustrates this finding. Additionally, the target genes predicted by all differentially expressed lncRNAs in the infected group were enriched in 117 signaling pathways compared to those in the adjacent group (ATT_vs_ADJ). KEGG signaling pathway enrichment analysis revealed that tight Junction, MAPK, endocytosis, and other signaling pathways exhibited a higher degree of gene enrichment, as depicted in Figure 2F.

### 2.5. Target Gene Identification and Functional Analysis of DE miRNAs

**Figure 3:**
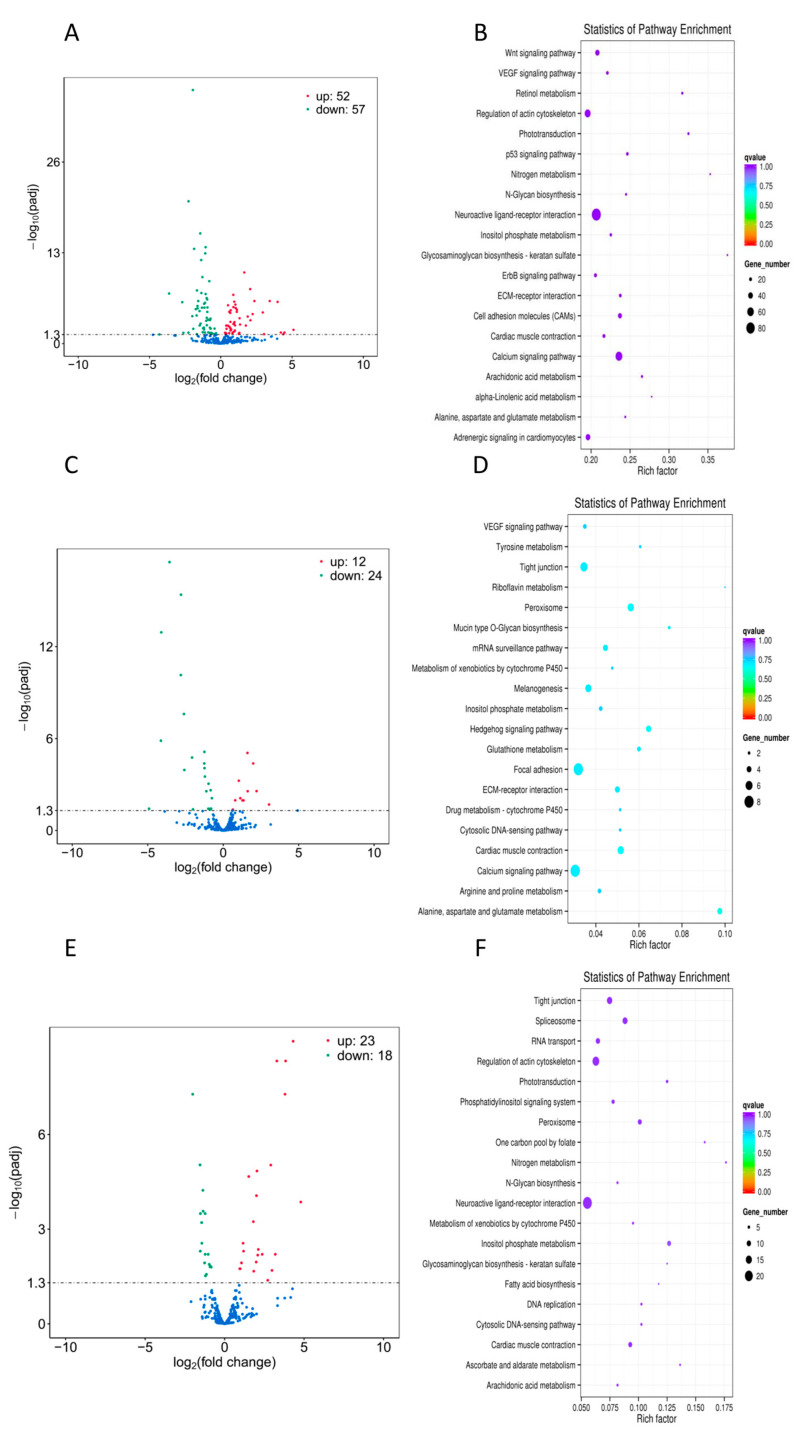
(**A**,**C**,**E**) Volcano plots from the differentially expressed miRNAs of different groups (ATT_vs_PER, ADJ_vs_PER, ATT_vs_ADJ). The −log10 of the *p*adj value is used for the Y axis and the log2 of the fold change is used for the X axis. The red dots are upregulated miRNAs and the green dots are downregulated miRNAs. The blue dots were non-significant differentially expressed miRNAs. (**B**,**D**,**F**) KEGG pathway analysis of target genes of the differentially expressed miRNAs of different groups (ATT_vs_PER, ADJ_vs_PER, ATT_vs_ADJ). The vertical coordinates depict the various pathways, while the horizontal coordinates indicate the proportion of genes that exhibit significant differential expression within each respective pathway relative to the total number of genes within that pathway. The size of the circles corresponds to the number of genes enriched within each pathway, with larger circles denoting a more significant enrichment. The color of the circles signifies the level of significance of the enrichment, with shades closer to red indicating a higher degree of significance.

In the reference genome of *T. ovatus*, a total of 1000 miRNAs were identified consisting of 520 known miRNAs and 480 novel miRNAs. In the present study, a comprehensive analysis of miRNAs and lncRNAs was conducted to investigate their differential expression patterns in three distinct groups, namely ATT_vs_PER, ADJ_vs_PER, and ATT_vsA_DJ. The results revealed the identification of 109, 36, and 41 miRNAs in the aforementioned groups, with 52, 12, and 23 miRNAs exhibiting upregulation. Furthermore, the analysis also revealed the downregulation of 57, 24, and 18 differentially expressed lncRNAs (Figure 3A,C,E). Three co-differentially expressed miRNAs were found in the ATT_vs_PER, ADJ_vs_PER, and ATT_vs_ADJ comparison groups through the analysis of differentially expressed miRNAs between groups.

The target genes anticipated by all miRNAs differentially expressed in the infection group (ATT_vs_PER) were enriched in 149 signaling pathways. An analysis of the KEGG signaling pathways demonstrated that several pathways, including neuroactive ligand-receptor interaction, calcium, MAPK, regulation of the actin cytoskeleton, endocytosis, and focal adhesion signal pathways, were enriched with more genes (Figure 3B). Similarly, the target genes predicted by all differentially expressed miRNAs in the infection-adjacent (ADJ_vs_PER) were enriched in 82 signaling pathways. The results of the KEGG signal pathway enrichment analysis indicate that more genes were enriched in the neuroactive ligand-receptor interaction, focal adhesion, and calcium signaling pathways, as illustrated in Figure 3D. Furthermore, the target genes predicted by all differentially expressed lncRNAs in the infected group were enriched in 124 signaling pathways, a higher number than those in the ATT_vs_ADJ group. The KEGG signaling pathway enrichment analysis showed that multiple genes were enriched in the neuroactive ligand-receptor interaction, actin cytoskeleton, endocytosis, MAPK, and tight junction signaling pathways, as shown in Figure 3F.

### 2.6. Construction and Visualization of the lncRNA–miRNA–mRNA Network

**Figure 4:**
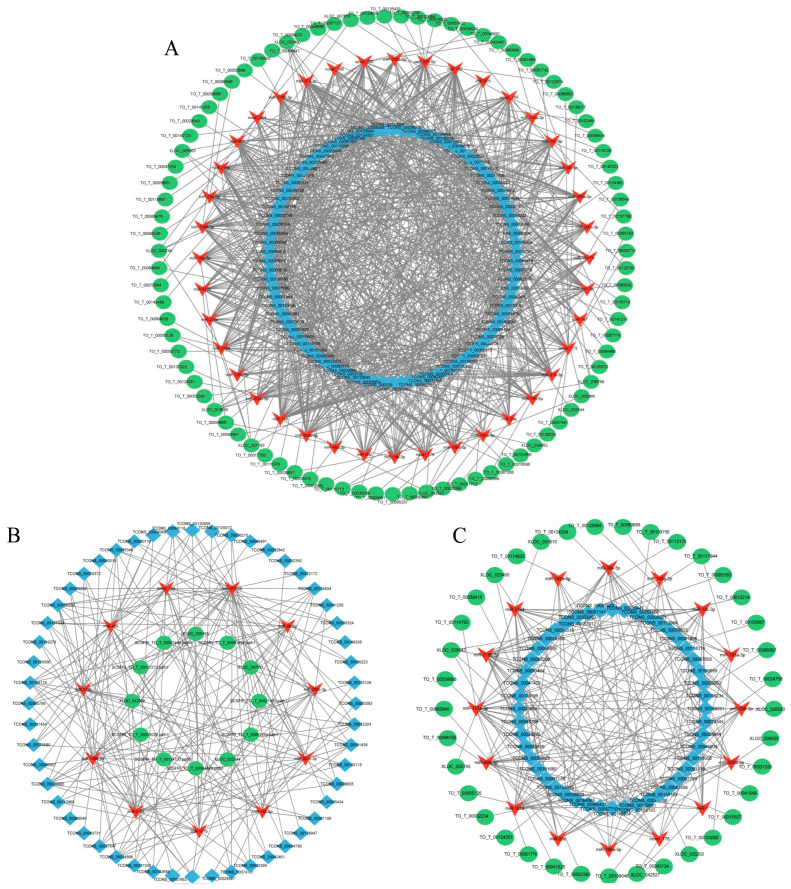
Identification of the lncRNA-miRNA-mRNA competing endogenous RNA (ceRNA) network. (**A**) Infection group (ATT_vs_PER), (**B**) infection-adjacent group (ADJ_vs_PER), and (**C**) infection group compared with infection-adjacent (ATT_vs_ADJ). The red “V” represents miRNA, the blue square represents lncRNA, and the green circular shape represents mRNA.

Based on the ceRNA theory, a pivotal lncRNA-miRNA-mRNA triple regulatory network was established, elucidating the significant contribution of non-coding RNAs in the immune resistance of *T. ovatus* against *C. irritans* infection. As mentioned above, the lncRNA-miRNA-mRNA network exhibited 213 nodes and 972 edges in the infection group, encompassing 40 miRNAs, 86 lncRNAs, and 87 mRNAs (Figure 4A). An examination of mRNA enrichment within the differential regulation network determined that various gene ontology (GO) items, including proteolysis, nitrogen compound transport, lysosome, proteasome, and pyrimidine metabolism, exhibited enrichment. KEGG pathways, such as phagosome protein processing in the endoplasmic reticulum, the toll-like receptor signaling pathway, and neuroactive ligand-receptor interaction, were identified. Furthermore, the expression of mir-218a was regulated by lnc-tcons_00055854 and lnc-tcons_00037053, which in turn affected the expression of the target gene *ga45a*.

The network in the infection-adjacent group (ADJ_vs_PER) comprises 101 nodes and 206 edges, with 16 miRNAs, 48 lncRNAs, and 37 mRNAs among the nodes (Figure 4B). The mRNA enrichment analysis revealed that the differential regulatory network was enriched with KEGG pathways such as proteasome, biosynthesis of amino acids, ribosome, and lysosome. The expression of mir-2188-5p is regulated, and the expression of target gene CDK4 is affected by lnc-tcons_00125045, lnc-tcons_00059833, and lnc-tcons_00005003. Similarly, the expression of mir-133A-3p is regulated, and the expression of the target gene PCKGM is affected by lnc-tcons_00093119, lnc-tcons_00059828, and lnc-tcons_00059826.

The network comprising 75 nodes and 196 edges was more prevalent in the infected group than in the adjacent group (ATT_vs_ADJ). The nodes encompassed 11 miRNAs, 52 lncRNAs, and 12 mRNAs, as depicted in Figure 4C. The differential regulatory network was analyzed for mRNA enrichment and found to be enriched in several KEGG pathways, including autophagy, cellular catabolic process, regulation of autophagy, regulation of cellular catabolic process, cellular component organization, cardiac muscle contraction, adrenergic signaling in cardiomyocytes, and neuroactive ligand-receptor interaction.

### 2.7. Validation of RNA-seq by qt-PCR

**Figure 5:**
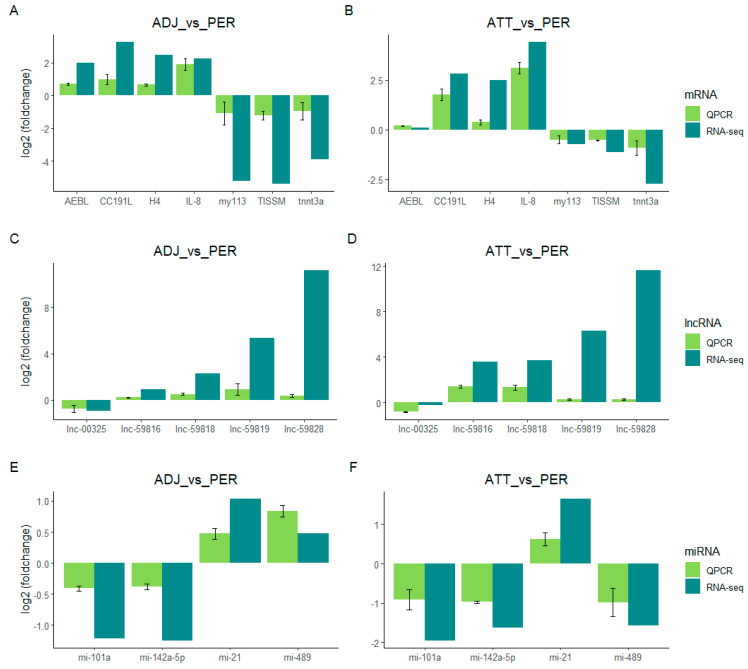
Validation of differentially expressed RNA by q–PCR. (**A**,**B**) Differentially expressed mRNA; (**C**,**D**) differentially expressed lncRNA; and (**E**,**F**) differentially expressed miRNA. The fold difference in the expression of qPCR was calculated from ΔΔCt.

In order to authenticate the precision of RNA-seq, a total of 7 differentially expressed genes (DEGs), 4 differentially expressed miRNAs (DEMs), and 5 differentially expressed lncRNAs (DELs) were randomly selected from the lncRNA-miRNA-mRNA network for quantitative real-time polymerase chain reaction (qt-PCR) analysis. This analysis aimed to determine the expression patterns of these genes in various treatment groups of skin tissue. The qt-PCR results demonstrated that the expression patterns of the selected genes in different treatment groups were fundamentally consistent with the RNA-seq analysis results (Figure 5). This finding confirms the specificity and accuracy of the transcriptome sequencing outcomes.

## 3. Discussion

The skin serves as the principal site for *C. irritans* infection and is the most extensive immunologically active organ in fish, serving as a crucial physiological barrier against *C. irritans* infection [20,21]. The spatial separation between each infected site on the skin and the variance in immune expression between uninfected and infected skin in *T. ovatus* trevally following *C. irritans* infection remains unclear. In recent times, a growing body of evidence indicates that non-coding RNAs (ncRNAs), such as miRNAs and lncRNAs, play crucial regulatory roles in gene expression networks, thereby influencing various biological processes, particularly immune regulation [22,23,24,25,26,27]. In order to gain a deeper understanding of the differences in innate immunity between uninfected and infected skin, we conducted a systematic comparison of the entire transcriptome of infected, adjacent, and infected versus adjacent skin (ATT_vs_PER, ADJ_vs_PER, ATT_vs_ADJ).

The examination of mRNA data revealed that 458 genes were differentially upregulated and 578 genes were differentially downregulated in the comparison of adjacent versus infected (ATT_vs_ADJ). Notably, the upregulated differential genes were significantly enriched in the cytokine-cytokine receptor interaction pathway, which plays a crucial role in intercellular regulation, cellular mobilization, innate and adaptive inflammatory host defense, cell growth, differentiation, cell death, and developmental and repair processes aimed at restoring endostasis [28,29]. The results of a comparative differential gene analysis demonstrated that the expression of CCL25 and its receptor CCR9s was upregulated in close proximity to *C. irritans* infection sites. In mammals, the CCL25/CCR9 pathway plays a crucial role in T-cell development and small intestinal immunity. Yang et al.’s study on orange-spotted grouper (*Epinephelus coioides*) revealed that during the late stage of *C. irritans* infection, the expression of CCL25 and its receptor CCR9 was elevated in immune tissues such as the head, kidney, spleen, and skin [30,31]. When CCL25 is rapidly expressed in an inflammatory site, effector cells are recruited to the inflammatory tissue, and the inflammatory response occurs. As a receptor of CCL25, CCR9 is expressed on the cell surface. When CCR9 binds to CCL25, it transmits cell signals and regulates cell-life activities. Expression was elevated, proving the CCL25/CCR9 complex may be involved in host defense against *C. iritans* infection. Comparative differential gene analysis revealed that the expression of interleukin 17 receptor A (*il17a*) was elevated in sites adjacent to the *C. iritans* infection. IL17 family cytokines induce the expression of inflammatory cytokines, chemokines, and antimicrobial proteins by forming dimers, and they must bind to IL17 receptors (*il17rs*) on the cell surface for signaling [32]. The SEFIR structural domain of IL17R proteins interacts with ubiquitin ligases upon binding to IL17 ligands, thereby initiating a signaling cascade that activates other inflammatory cytokines and chemokines associated with the immune response [33]. Wang et al. observed an upregulation of *il17ra* expression in the intestinal tissues of channel catfish (*Ictalurus punctatus*) during the later stages of bacterial infection, highlighting the crucial role of mucosal immunity mediated by IL17 family cytokines in pathogen defense [34].

This study aimed to investigate the involvement of lncRNA and miRNA in skin resistance to *C. irritans* infection. A comparison was made between parasite-infected and adjacent uninfected skin sites to achieve this. The differential expression patterns of lncRNAs were analyzed in the infected group (ATT_vs_PER) and the adjacent group (ADJ_vs_PER), identifying 210 and 180 differentially expressed lncRNAs, respectively. The enrichment analysis of the target gene for the differentially expressed lncRNAs demonstrated that most genes were enriched in tight junction, MAPK, cell adhesion molecules (CAMs), and toll-like receptor signaling pathways. Previous research has shown that siganus oramin (*Siganus canaliculatus*) and large yellow croaker (*Larimichthys crocea*) were enriched in these pathways following *C. irritans* infection with differential genes [35,36]. Our study identified an upregulation of phosphoinositide-3-kinase regulatory subunit 3b (*pi3kr3b*) in the toll-like receptor signaling pathway. The *pi3kr3b* encodes a regulatory subunit of pi3k that binds activated protein tyrosine kinases to regulate their activity through two SH2 domains. Phosphoinositide-3-kinase (*pi3k*) is a crucial player in various cellular processes, including cell survival and differentiation, by phosphorylating phosphatidylinositol family members on the cell membrane and initiating downstream target genes to generate signaling cascades [37,38]. In Nile tilapia (*Oreochromis niloticus*) and largemouth bass (*Micropterus salmoides*), pi3k expression was observed to increase in immune organs such as the head and kidney following bacterial infection, indicating its potential immune-related functions [39,40]. This study observed a reduction in the lncRNAs expression associated with *pi3kr3b* (tcons_00060612) following *C. irritans* infection. It is postulated that tcons_00060612 plays a role in regulating pi3kr3b expression.

**Figure 6:**
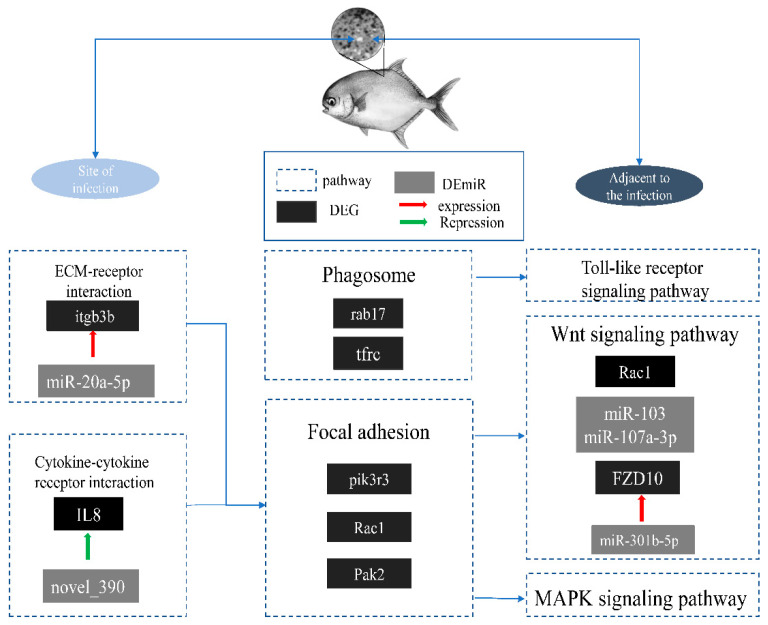
A schematic diagram of the predictive molecular mechanism of *C. irritans* infection in *T. ovatus*. Key DEmiR, DEGs, and pathways in the skin tissue of *T. ovatus* during stimulation of *C. irritans* infection.

Furthermore, differential analysis of miRNA expression patterns was conducted across the infected group (ATT_vs_PER), the infection-adjacent group (ADJ_vs_PER), and the infected group relative to the infection-adjacent group (ATT_vs_ADJ). KEGG enrichment analysis was performed on the target genes of the differentially expressed miRNAs. The enrichment analysis results indicate that differential miRNAs were enriched in the p53, Wnt and VEGF signaling pathways (Figure 6). Specifically, the miR-142a-3p, miR-103, miR-489, and miR-27b-3p target genes were enriched in these pathways. These findings suggest that miRNAs act as signaling regulators and may play a role in controlling inflammatory responses. Fan et al.’s study revealed that miR-142a-3p is implicated in the immune regulation of grass carp (*Ctenopharyngodon idella*) [41]. The expression of miR-142a-3p was downregulated following grass carp infection with *Aeromonas hydrophila*. This microRNA was found to reduce cell viability and stimulate apoptosis by regulating target genes, and it serves multiple functions in the host’s antimicrobial immune response. Dong et al.’s research demonstrated that miR-103 plays a crucial role in regulating the IL-1R1 gene, which is involved in the immune and inflammatory responses of Miiuy croaker (*Miichthys miiuy*) [42]. IL-1R1 is a member of the IL-1 family. IL-1 induces the conformational change of IL-1R1, which binds to the IL-1 receptor accessory protein (IL-1RAcP). As IRAK is recruited to the receptor complex, the complex is split, and finally, the NF-κB, JNK and p38 signaling pathways are activated, which results in the expression of inflammatory cytokines. In the study, Gao et al. discovered that miR-489 functions as a negative regulator that inhibits the activation of NF-κB, IL-8, and ISRE reporter genes via TRAF6. TRAF6 is a critical member of the TNFR family and a key adapter for the toll-like / interleukin-1 receptor superfamily. It can directly bind to the interleukin receptor-associated kinase, activating the transcription factor nuclear factor-κB (NF-κB), activating a series of inflammatory cells, and releasing inflammatory factors. This regulatory mechanism helps to maintain immune system stability in miiuy croaker by downregulating TRAF6 expression, thereby preventing the onset of autoimmune diseases [43]. Additionally, exposure of carp (*Cyprinus carpio*) to bisphenol A results in the downregulation of miR-27b-3p expression, which in turn regulates CYP1B1 expression and induces oxidative stress through the mitochondrial pathway, ultimately leading to apoptosis in carp splenic lymphocytes [44].

The ceRNA network was established using differential expression analysis across various comparison groups. The targeting relationships of the distinct RNA molecules that exhibited differential expression between groups were examined, and GO and KEGG analyses were conducted on the ceRNA network to predict the potential function of miRNA-regulated differentially expressed mRNA. This approach led to identifying several novel targets in crucial immune pathways. The enrichment outcomes underscored the significance of the toll-like receptor signaling pathway. TLRs are primarily responsible for facilitating the production and secretion of cytokines, which in turn elicit inflammatory responses. Specifically, PAMPs from pathogens activate the TLRs expressed on the surface of monocyte-macrophages (MMs), which subsequently activate intracellular signaling pathways. This activation leads to the translocation of NF-κB into the nucleus, thereby initiating the transcription of pertinent genes and corresponding mRNAs. The process above results in the production and secretion of various cytokines, including but not limited to IL-1, IL-6, IL-8, IL-12, TNF-α, and IFN-γ, into the extracellular milieu. This event triggers the accumulation and convergence of granulocytes and macrophages, thereby initiating an early immune response [45,46,47,48,49]. Li et al. conducted a study that confirmed the upregulation of IL-8 expression in the skin of orange-spotted grouper (*Epinephelus coioides*) infected with *C. irritans*, indicating its potential role in the resistance of grouper to *C. irritans* infection [50]. IL-8 is one of the earliest CXC chemokines found in fish. It can be produced by macrophages, vascular endothelial cells, epithelial cells, and other types of cells and recruit neutrophils to inflammatory sites. This study demonstrates an increase in Interleukin-8 (IL-8) expression in skin tissues after *C. irritans* infection. The ceRNA network constructed in this investigation reveals the potential involvement of the newly predicted miRNA novel_390 in regulating IL-8 expression, thereby influencing the early immune response following *C. irritans* infection in *T. ovatus*.

## 4. Materials and Methods

### 4.1. Cryptocaryon irritans and Experimental Fish

*C. irritans* were derived from naturally infected *T. ovatus*, and 150 *T. ovatus* (180 ± 10 g) were used as the host to establish the passage system. Theront propagation and tomont collection were conducted following the method of Dan et al [51]. The *T. ovatus* were infected with a safe concentration of theronts (8000 theronts/fish). During the infection process, the density of *T. ovatus* was 5 L of seawater per fish, which were maintained in the dark for 2 h. Then, the infected *T. ovatus* were transferred to a new container without theronts for feeding. Feeding was perfomed by way of micro running seawater and blowing sufficient air into the seawater. After 4 days, a large number of tomonts was found to be attached to the bottom of the container, and the *T. ovatus* were again transferred to a new container, ready to start the next infection. The water in the container in which the tomonts were attached was drained, and the tomonts were collected into a beaker by the siphon method. After the tomonts were cleaned, they were pumped into air and the theronts were left to hatch.

*T. ovatus* was obtained from the Shenzhen base of the South China Sea Fisheries Research Institute, Shenzhen City, Guangdong Province, China. Before the experiment, the experimental fish were acclimated to the water tank (DO 8.8 ± 0.2 mg/L, pH 8.1–8.3) for 1 month. Fish were fed twice a day during the acclimation period with compound feed weighing 5% of their body weight. Feeding ceased the day before the start of the experiment.

### 4.2. Experimental Methods and Sample Collection

The study was structured into two distinct groups, the infection group, and the control group, each consisting of three parallel groups containing ten fish. The method of *C. irritans* infection was the same as described in Section 4.1. The skin tissues of three fish were collected from each parallel group of both the infection and control groups. The skin tissues of the control group were observed to be uninfected (PRE), while those of the infection group were segregated into two distinct areas, namely the theronts attachment area (ATT) and adjacent area (ADJ). After rapid freezing with liquid nitrogen, the skin tissue was stored at −80 °C until RNA extraction.

### 4.3. Total RNA Extraction, Library Construction, and Illumina Sequencing

Following RNA extraction protocols, the total RNA of the *T. ovatus* skin tissue was extracted. The total RNA integrity was determined by electrophoresis on 1% agarose gel. The NanoDrop-2000 ultraviolet imaging system detected the concentration of RNA and the OD260/OD280 ratio.

After the total RNA was tested for qualification, the small RNA sample pre-kit was used to construct the library. Using the special structure of the small RNA’s 3′ and 5′ ends, the total RNA was used as the starting sample, and the cDNA was reverse transcribed from small RNA by directly splicing both ends. Then, the target DNA fragments were amplified by PCR and separated by polyacrylamide gel electrophoresis. Cutting gel and recycling were used to obtain the cDNA library.

LncRNA library was constructed by removing ribosomal RNA. First, ribosomal RNA was removed from the total RNA, and then RNase R was used to break it into short fragments of 250~300 bp. Using the RNA fragment as a template, a random oligonucleotide primer was used to synthesize the first cDNA strand. The RNA strands were degraded with RNase H. Under the DNA polymerase I system, dNTPs were synthesized into the second strand of cDNA. The purified double-stranded cDNA was repaired at the end, a tail was added, and sequenced. The second strand of cDNA containing U was degraded by the USER (uracil-specific excision reagent) enzyme, and PCR amplification was performed to obtain the library.

### 4.4. Data Analysis and Quality Control

The raw data (raw reads) of the fastq format were checked with Fastp v0.23.4 [52]. In order to obtain clean reads, the following reads were removed: (1) reads with a 5′ adapter, 3′ adapter, or insert sequence; (2) reads with more than 10% N; (3) reads with more than 50% nucleotides with Qphred ≤ 20; and (4) reads with ploy A/T/G/C. The adapter sequences were also trimmed from the 3’ end of the reads. At the same time, the clean data’s Q20, Q30, and GC content were calculated. Clean data with high quality were used for all downstream analyses.

### 4.5. mRNA Differential Expression and Enrichment Analysis

This study measured gene expression levels using FPKM (fragments per kilobase of exon model per million mapped reads) [53]. DESeq2 v1.40.2 [54] software was used to identify differentially expressed genes (DEGs) which satisfied *p* < 0.05 and |log2 (FoldChange)| > 1. In organisms, different genes coordinate to perform their biological functions. Therefore, the analysis based on specific signaling pathways is helpful in further understanding the biological function of the gene pathway. The main public pathway database is KEGG (Kyoto Encyclopedia of Genes and Genomes). The Clusterprofiler v4.8.1 [55] was used for KEGG pathway enrichment analysis, and a hypergeometric test was applied to find the pathway of the significant enrichment of the differentially expressed genes.

### 4.6. LncRNA and miRNA Identification

Clean reads were mapped to the genome of *T. ovatus* using HISAT2 v2.2.1 [56] and assembled using StringTie v2.2.1 [57]. LncRNAs were identified from the assembled transcripts as follows: (1) the transcripts with low expression were removed by FPKM < 0.5; (2) transcripts with a length greater than 200nt and number of exons ≥ 2; (3) CNCI, Pfam, and CPC2 databases were used to remove transcripts with protein-coding ability; (4) Cuffcompare removed the transcripts of annotated genes within a 1 KB flanking region.

To identify conserved miRNAs, MirDeep2 v2.0.1.2 was used to compare predicted miRNA hairpins against miRNA precursor sequences from miRBase22.0 [58], and srna-tools-cli was used to obtain the potential miRNA and draw its secondary structure. The hairpin structures of miRNA precursors were used to predict novel miRNAs. The available miREvo v1.2 [59] and mirdeep2 v2.0.1.2 software was integrated to predict novel miRNAs based on secondary structures.

### 4.7. Differential Expression and Enrichment Analysis

LncRNA and miRNA were quantified using StringTie software, and the transcription of reads/kilobase (RPKM) per million reads mapped was obtained. EdgeR v3.42.4 [60] software was used for differential expression analysis. The *p* values obtained were controlled for the error detection rate using the method of Benjamin and Hochberg. LncRNA and miRNA with |log2 (fold change)| > 0 and *p*adj < 0.05 were assigned as differentially expressed.

Using clusterprofiler v4.8.1 R packages, GO and KEGG enrichment analysis was conducted on the target genes of differentially expressed lncRNAs and miRNAs. Enrichment was considered significant when the corrected *p* value was less than 0.05.

### 4.8. Competing Endogenous RNA (ceRNA) Network Construction

Based on the ceRNA theory, we searched for lncRNA target gene pairs with the same miRNA binding sites and constructed a regulatory relationship with lncRNA as the bait, miRNA as the core, and mRNA as the target. At the whole transcriptome level, the mechanism of ncRNA regulation of gene expression was revealed by the ceRNA regulatory network. Based on the obtained ceRNA regulatory networks, interaction network maps were constructed and visualized using Cytoscape v3.10.1 software.

### 4.9. Quantitative Real-Time PCR (qPCR) Verification

To assess the dependability of the transcriptome sequencing data, a total of eight differentially expressed mRNAs, six differentially expressed lncRNAs, and four differentially expressed miRNAs were chosen for validation through qPCR. The Primer Premier v5.0 software was utilized to design primers for mRNA and lncRNA, with the specific primer sequences presented in Table S1. The stem-loop method was employed to design miRNA reverse transcription primers, with the stem ring general sequence for GTCGTATCCAGTGCAGGGTCCGAGGTATTCGCACTGGATACGAC and downstream universal primers for GCAGGGTCCGAGGTATTC. The specific primer sequences of miRNA were presented in Table S2. Employing EF as the reference gene, the fluorescence quantitative PCR was utilized to measure the expression levels of randomly chosen mRNA, lncRNA, and miRNA. The 2^−ΔΔCt^ method was employed to determine the relative expression levels of the target genes with the reference gene.

## Figures and Tables

**Table 1:** Summary of the transcriptome of *T. ovatus*.

Sample_Name	Raw_Reads	Clean_Reads	Error Rate (%)	Q20 (%)	Q30 (%)	GC_Content (%)	Total Mapped (%)
ADJ_1	82,430,898	81,556,240	0.03	97.41	93.01	47.74	86.81
ADJ_2	83,301,486	82,361,856	0.03	97.53	93.3	50.48	84.03
ADJ_3	82,206,822	81,388,816	0.03	97.25	92.65	46.61	89.64
ATT_1	81,229,824	80,361,428	0.03	97.2	92.58	46.69	85.75
ATT_2	83,299,108	82,251,852	0.03	96.93	92.12	46.41	86.45
ATT_3	80,793,446	79,959,968	0.03	97.3	92.8	46.67	88.20
PRE_1	81,092,584	80,206,522	0.03	97.1	92.41	48.72	85.43
PRE_2	85,308,302	84,316,544	0.03	97.32	92.93	47.53	85.69
PRE_3	93,181,024	91,990,894	0.03	97.05	92.45	46.79	84.21

## Data Availability

The data presented in this study is available on request from the corresponding author.

## Supplementary Materials

The following supporting information can be downloaded at: https://www.mdpi.com/article/10.3390/ijms242115886/s1.
