# Supplementary material for: Transcriptomic Analysis Reveals Functional Interaction of mRNA-lncRNA-miRNA in Trachinotus ovatus Infected by Cryptocaryon irritans
Source: Int J Mol Sci. 2023 Nov 1;24(21):15886. doi: 10.3390/ijms242115886 (PMC10648848; doi:10.3390/ijms242115886)
Supplement: Supplementary file 1 [file ijms-24-15886-s001.zip › ijms-2604749-supplementary.pdf]

Table S1. Sequences of primer sets used in this study.

RNA Type	Primer Name	Sequence(5'to3')
mRNA	IL8-F	TCATTGCTGTGGTGGTGCT
	IL8-R	GCCTGTCTTTTTCAGAGTGG
mRNA	CC19lL-F	CAGGGACACTGGCGGAGAT
	CC19lL-R	GGGTTCGTTAGCAGGCACA
mRNA	tnnt3a-F	GGATTGAGAAGCGTCGTGC
	tnnt3a-R	CAGGTGACTGCTGTAGTTGGAG
mRNA	myosin7-F	GGAGCAGAAAAAAAGCAGTG
	myosin7-R	GCCAAGATGAACATTAGCCT
mRNA	TISSM-F	CAAGTTCAAAAAGCCTGCC
	TISSM-R	GCCTCCACATTCTTACGCC
mRNA	myl13-F	GCCAGAATCCCACCAATGC
	myl13-R	ACACCCTCAGACCCTCCACA
mRNA	AEBL-F	ACAAGGACACCGAGGGGAT
	AEBL-R	GCCTGGCTCTTCAGTATGGA
lncRNA	Lnc-59819-F	CTGTCTGTTTCTTTTCGTCC
	Lnc-59819-R	CCTGTTCCTCATCTTCTCTCT
lncRNA	Lnc-59818-F	GCCTTCAGTCTGTCTGTTTCTT
	Lnc-59818-R	GCCTGTTCCTCATCTTCTCTC
lncRNA	Lnc-59828-F	GGAAAGACAGGAAGTAACACG
	Lnc-59828-R	AGACAGACTGAAGGCGGAT
lncRNA	Lnc-59816-F	CCTTCAGTCTGTCTGTTTCTT
	Lnc-59816-R	GCCTGTTCCTCATCTTCTC
lncRNA	Lnc-00325-F	GTTTGCTGGTGGTCGTGTT
	Lnc-00325-R	GGTTTTGGGTATTCTTGTCTCT
microRNA	Mir-101a-F	CGGGCTACAGTACTGTGATA
microRNA	Mir-21-F	CGGGCTAGCTTATCAGACTGG
microRNA	Mir-489-F	CGGGCAGTGACATCATATGTA
microRNA	Mir-142a-5p-F	CGGGCCATAAAGTAGAAAG
microRNA	UPM-R	CAGCCACAAAAGAGCACAAT

Table S2. Sequences of stem-loop RT primer sets used in this study.

Primer Name	Sequence(5'to3')
Mir-101a-RT	CCTGTTGTCTCCAGCCACAAAAGAGCACAATATTTCAGGAGACAACAGGCTTCAGT
Mir-21-RT	CCTGTTGTCTCCAGCCACAAAAGAGCACAATATTTCAGGAGACAACAGGGCCAACA
Mir-489-RT	CCTGTTGTCTCCAGCCACAAAAGAGCACAATATTTCAGGAGACAACAGGGCAGCCG
Mir-142a-5p-RT	CCTGTTGTCTCCAGCCACAAAAGAGCACAATATTTCAGGAGACAACAGGAGTAGTG
